# Vascular disease in cancer: Current and emerging concepts^☆^

**DOI:** 10.1016/j.ahjo.2022.100143

**Published:** 2022-05-21

**Authors:** Nausheen Akhter

**Affiliations:** Northwestern University, United States of America

**Keywords:** Vascular complications, cancer, Cardio-oncology

Cardiovascular toxicities in patients with cancer have focused mainly on cancer therapy-related cardiac dysfunction. Vascular disease was not considered a primary concern. However, adverse vascular events are very common in the oncology population due to a triad of overlapping risk factors: exposure to vascular-toxic anti-cancer therapies, increased arterial and venous thromboembolic event (VTE) risk from cancer itself, and predisposing clinical and genetic risk factors.

As a first part of the triad, a wide array of anti-cancer therapies (Table 1) used in both solid and hematologic malignancies contribute to vascular and metabolic complications, including hypertension (HTN), atherosclerosis, coronary artery disease (CAD), peripheral arterial disease, cerebral vascular disease, pulmonary arterial hypertension, and thromboembolic events (Fig. 1). Cisplatin is an alkylating agent with anti-tumor effects for testicular, head and neck, bladder, melanoma, lymphoma, and gynecologic malignancies. It can cause HTN, Raynaud's phenomenon, VTE, dyslipidemia, and CAD. Cisplatin-based regimens have a two-fold increased risk of thromboembolic events compared to oxaliplatin-based regimens [1].

**Table 1:** Drug class, cardiovascular toxicity and preventative measures.

Drug class	Major adverse cardiovascular effects	Preventative measures^a^
Alkylating agents	HTN, HL, CAD, VTE, Raynaud's	Blood Pressure surveillance Glucose surveillance Lipid surveillance Thromboprophylaxis in high risk
Immunomodulatory	VTE	Thromboprophylaxis in high risk
Antiangiogenic (VEGF-Inhibitors)	HTN, ATE, CTRCD	Blood Pressure surveillance Thromboprophylaxis in high risk ECG surveillance Echocardiogram surveillance
Antimetabolites	Vasospasm, ischemia	Peri-infusion of anti-anginal therapy Switch from continuous to bolus infusion
Anthracycline	Impaired coronary reactivity, CTRCD	Baseline Echocardiogram Echocardiogram after 250 mg/m^2^ and every additional 100 mg/m^2^ doxorubicin equivalent dose Echocardiogram at 6–12 months
BCR-ABL Tyrosine Kinase Inhibitors	CAD, PAD, CVA, pulmonary HTN	Blood Pressure surveillance Glucose surveillance Lipid surveillance Ankle-brachial index ECG surveillance Echocardiogram

Hypertension (HTN), hyperlipidemia (HL), coronary artery disease (CAD), venous thromboembolic event (VTE), arterial thromboembolic event (ATE), cancer therapy related cardiac dysfunction (CTRCD), electrocardiogram (ECG), peripheral arterial disease (PAD), cerebral vascular attack (CVA).

a Counsel lifestyle preventative measures including diet and exercise modification, and tobacco cessation.

**Fig. 1:**
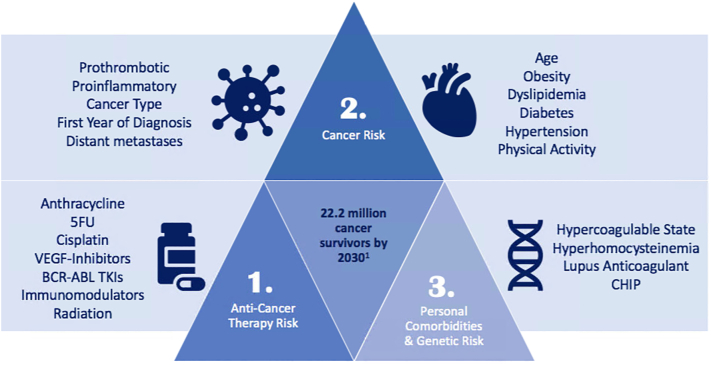
The triangle of cancer, chemotherapy, and vascular insult. A triad of risk factors contribute to vascular complications in cancer survivors. First, a wide array of anti-cancer therapies used in both solid and hematologic malignancies contribute to vascular and metabolic complications. Second, cancer itself contributes to an increased risk through multiple mechanisms. Third, personal comorbidities and emerging genetic risk factors must be considered.

Immunomodulatory therapies such as thalidomide, lenalidomide, and pomalidomide have been shown to block several pathways for disease progression in multiple myeloma. In combination with high-dose steroids or cytotoxic chemotherapy regimens, patients on immunomodulatory therapies with >1 clinical risk factor, such as prior VTE, erythropoietin-stimulating agents, diabetes, CAD, and chronic kidney disease, are at increased risk for VTE. Starting thromboprophylaxis with aspirin, prophylactic/therapeutic low molecular weight heparin, or warfarin is recommended depending on the patient's clinical risk [2].

Bevacizumab is a monoclonal antibody against the vascular endothelial growth factor receptor (VEGFR), which inhibits angiogenesis and has adverse vascular effects, including HTN and arterial thromboembolism (ATE)/VTE. In a meta-analysis of 13,026 patients from 20 randomized trials, the relative risk of ATE with bevacizumab-based therapies vs. controls was 1.46 (95% CI: 1.11–1.93, *p* = 0.007) [3].

There are several well-recognized vascular toxicities associated with fluorouracil (5-FU), including ischemia and coronary vasospasm, which work through either direct cellular damage or indirectly through endothelial dysfunction. In a single-center study of 4019 patients treated with 5-FU, the incidence of coronary vasospasm was 2.16% and was more often seen in younger patients with less traditional cardiovascular risk factors [4]. Generally, re-challenging patients with 5-FU reproduces chest pain symptoms. Therefore, this should be considered with caution. Vascular toxicities may be ameliorated by switching from continuous to bolus infusion and treating patients with prophylactic anti-anginal agents, calcium channel blockers, and nitrates [5]. Impaired coronary vasoreactivity is a novel mechanism for early doxorubicin cardiotoxicity. In a study on canines who received doxorubicin, coronary diameters were measured on stress-rest computed tomographic angiography, demonstrating impaired coronary vasoreactivity before a reduction in left ventricular ejection fraction is noted [6].

Radiation therapy can have latent coronary and non-coronary vascular effects such as in Hodgkin's lymphoma survivors who received high-dose chest and neck radiation. It is important to counsel survivors regarding the risks of radiation therapy, screening, and risk reduction strategies. There may be a role for agents such as statins, colchicine, and aspirin in preventing radiation-associated cardiovascular effects [7].

BCR-ABL tyrosine kinase inhibitors (TKIs) have revolutionized the treatment of patients with chronic myeloid leukemia; however, second and third-generation TKIs have significant vascular and metabolic effects. Dasatinib causes pulmonary hypertension. Nilotinib causes hyperglycemia and atherosclerosis. Ponatinib causes HTN and vascular disease. Thrombotic microvascular angiopathy is a potential mechanism for cardiotoxicity from ponatinib. A murine model of ponatinib-cardiotoxicity noted segmental wall motion abnormalities and patchy perfusion defects despite normal coronaries [8].

The second part of the triad leading to adverse vascular risk is cancer itself, which is a pro-thrombotic state that can lead to increased venous and arterial thrombotic events. Approximately 20% of the overall VTE burden is in patients with cancer [9]. The rate of first VTE was seven-fold higher in patients with cancer than those without cancer, with the highest risk of VTE occurring within the first year of diagnosis [10]. The presence of distant metastases increases the risk of VTE across most cancer subtypes [11]. The Khorana score (Table 2) was developed to risk-stratify patients with cancer (except those with brain tumors or myeloma) for VTE. It is comprised of five predictors: cancer type, pre-chemotherapy platelet count ≥350 K, hemoglobin level <10 g/dL or using RBC growth factors, pre-chemotherapy leukocyte count >11 K, and BMI ≥35 kg/m^2^. Two pivotal trials used the Khorana risk score with rivaroxaban and apixaban. High-risk ambulatory patients with cancers with a score of ≥2 were randomized to drug or placebo as prophylactic anticoagulation for VTE during systemic chemotherapy. In the CASSINI trial, 10 mg rivaroxaban did not result in a significantly lower incidence of VTE or death due to VTE in 180 days [12]. The AVERT trial demonstrated a significantly lower VTE rate with 2.5 mg of apixaban twice daily, although there was a higher rate of major bleeding with apixaban vs. placebo [13]. Therefore, the current American Society of Clinical Oncology guidance is that routine thromboprophylaxis should not be offered to all patients with cancer. It may, however, be considered in a select subset of high-risk patients who are not at increased risk for bleeding.

**Table 2:** Khorana score for risk of VTE in patients with cancer^a^.

Variable	Points
Cancer type	
Stomach	+2
Pancreas	+2
Lung	+1
Lymphoma	+1
Gynecologic	+1
Bladder	+1
Testicular	+1
Other	0
Pre-chemotherapy platelet count ≥350 × 10^9^/L	+1
Hemoglobin level <10 g/dL or using RBC growth factors	+1
Pre-chemotherapy leukocyte count >11 × 10^9^/L	+1
BMI ≥35 kg/m^2^	+1

a Not to be used with brain tumors or myelomas.

The third part of the triad is that of common clinical and genetic risk factors associated with CVD, VTE, and ATE in patients with cancer and CVD. Several patient-related factors have been associated with VTE in patients with cancer (Table 3) [14]. There are also genetic factors associated with VTE and ATE, such as hypercoagulable states: antithrombin, protein C or protein S deficiency, factor V Leiden, factor II G20210A, lupus anticoagulant, and hyperhomocysteinemia [15]. Clonal hematopoiesis of indeterminate potential (CHIP) is a pre-malignant state where clonal progeny in the hematopoietic stem cells of the bone marrow carry genetic mutations that increase the risk for both malignancy and CVD. There are four mutations (DNMT3A, ASXL1, TET2, JAK2) which account for the majority of mutations that increase adverse cardiovascular risk. CHIP carries a four-fold increased cardiovascular risk. Clinical management of patients with CHIP includes aggressive cardiovascular risk factor modification, with future study of anti-inflammatory therapies and treatment to reduce clones [16].

**Table 3:** Cancer and clinical variables associated with venous thromboembolism (VTE).

Cancer variables
Cancer Types: Myeloma, Stomach, Pancreatic, Lung, Lymphoma, Gynecologic, Bladder, Testicular
Timing: Within the first year of diagnosis
Stage: Presence of distance metastases

**General clinical variables**
Elderly patients (increasing by decade)
Immobility: surgery, pathologic fractures, frailness
Prior history of VTE
Comorbidities: obesity, infection, pulmonary and renal disease

## Declaration of competing interest

The authors declare that they have no known competing financial interests or personal relationships that could have appeared to influence the work reported in this paper.
